# Early ART initiation during infancy preserves natural killer cells in young European adolescents living with HIV (CARMA cohort)

**DOI:** 10.1002/jia2.25717

**Published:** 2021-07-08

**Authors:** Margherita Doria, Sonia Zicari, Nicola Cotugno, Sara Domínguez‐Rodríguez, Alessandra Ruggiero, Giuseppe R Pascucci, Alfredo Tagarro, Pablo Rojo Conejo, Eleni Nastouli, Kathleen Gärtner, Mark Cameron, Brian Richardson, Caroline Foster, Sion L Williams, Stefano Rinaldi, Anita De Rossi, Carlo Giaquinto, Paolo Rossi, Savita Pahwa, Paolo Palma, for the EPIICAL consortium

**Affiliations:** ^1^ Research Unit of Primary Immunodeficiency Bambino Gesú Children's Hospital IRCCS Rome Italy; ^2^ Research Unit of Clinical Immunology and Vaccinology Academic Department of Pediatrics (DPUO) Bambino Gesù Children's Hospital IRCCS Rome Italy; ^3^ Department of Systems Medicine Chair of Pediatrics University of Rome "Tor Vergata" Rome Italy; ^4^ Pediatric Infectious Diseases Unit Fundación para la Investigación Biomédica del Hospital Madrid Spain; ^5^ Department of Virology University College Hospital London UK; ^6^ Institute of Child Health University College London UK; ^7^ Department of Epidemiology and Biostatistics Case Western Reserve University Cleveland OH USA; ^8^ Imperial College Healthcare NHS Trust London UK; ^9^ Department of Microbiology and Immunology University of Miami Miller School of Medicine Miami FL USA; ^10^ Section of Oncology and Immunology Department of Surgery, Oncology, and Gastroenterology Unit of Viral Oncology and AIDS Reference Center University of Padova Padova Italy; ^11^ Istituto Oncologico Veneto (IOV)‐IRCCS Rome Italy; ^12^ Department of Mother and Child Health University of Padova Padova Italy

**Keywords:** cohort studies, HIV care continuum, immunology, paediatrics, ARV, viral suppression

## Abstract

**Introduction:**

HIV infection causes pathological changes in the natural killer (NK) cell compartment that can be only partially restored by antiretroviral therapy (ART). We investigated NK cells phenotype and function in children with perinatally acquired HIV (PHIV) and long‐term viral control (five years) due to effective ART in a multicentre cross‐sectional European study (CARMA, EPIICAL consortium). The impact of age at ART start and viral reservoir was also evaluated.

**Methods:**

Peripheral blood mononuclear cells (PBMCs) from 40 PHIV who started ART within two years of life (early treated patients (ET), ≤6 months; late treated patients (LT), > 6 months), with at least five years of HIV‐1 suppression (<40 HIV copies/mL), were collected between November 2017 and August 2018. NK phenotype and function were analysed by flow cytometry and transcriptional profile of PBMCs by RNA‐Seq. HIV‐1 DNA was measured by real‐time polymerase chain reaction (Data were analysed by Spearman correlation plots and multivariable Poisson regression model (adjusted for baseline %CD4 and RNA HIV viral load and for age at ART start as an interaction term, either ET or LT) to explore the association between NK cell parameters and HIV reservoir modulated by age at ART start.

**Results:**

A significantly higher frequency of CD56^neg^ NK cells was found in LT compared with ET. We further found in LT a positive correlation of CD56^neg^ NK cells with HIV‐1 DNA. LT also displayed increased expression of the NKG2D and NKp46 activating receptors and perforin compared with ET. Moreover, CD107a^+^ and IFN‐γ^+^ frequencies in non‐stimulated NK were associated with HIV‐1 DNA in LT patients. Finally, RNA‐Seq analysis showed in LT an up‐regulation of genes related to NK‐activating pathways and susceptibility to apoptosis compared with ET.

**Conclusions:**

We show that early initiation of ART during infancy preserves the NK compartment and is associated with lower HIV‐1 reservoir. Such condition persists over adolescence due to long‐term viral control achieved through effective ART.

## 1 Introduction

HIV‐1‐induced immunological impairment begins during acute infection and is only partially restored in children with perinatally acquired HIV (PHIV) if antiretroviral therapy (ART) is introduced during the chronic phase of infection. On the other hand, early initiation of ART preserves adaptive immune function [1, 2], decreases immune‐ageing process and viral diversity, with consequent reduction of the viral reservoir [3, 4, 5] and delay in disease progression. In particular, Ag‐specific CD4+ T cells and HIV antibodies (Ab) were significantly different in patients with distinct age at ART start, with the late treated (LT, >6 months) presenting with persistent HIV‐specific adaptive immunity compared to the early treated (ET, <6 months) [6, 7, 8, 9]. What remains to be defined is whether the timing of ART initiation affects efficient anti‐viral activity in the innate immunity, mainly with regard to the effector natural killer (NK) cells, which are known to play an important role in controlling HIV‐1 infection and containing the viral reservoir [10, 11].

NK can be classified in three major subsets based on their differential expression of CD56 and CD16: CD56^bright^ (CD3‐CD56^bright^CD16^−/low^) able to release relevant amounts of antiviral cytokines; CD56^dim^ (CD3‐CD56^dim^CD16+), which are highly cytolytic; CD56^neg^ (CD3‐CD56^neg^CD16+), functionally anergic, expanded in infectious diseases such as HIV [12]. Several studies defined the relevance of NK in HIV control in adults, however scarce data are available in PHIV. In particular, recent studies showed that NK cells maintain the killing capacity of resting HIV‐1‐infected T cells, either generated by experimental models of latency or derived from ART patients, upon virus reactivation [13, 14, 15, 16, 17]. Furthermore, few studies in patients spontaneously controlling viral replication independently showed the presence of an NK population with enhanced *ex vivo* responses that inversely correlated with HIV‐1 reservoir size [18, 19, 20]. Finally, an association was shown between cell activation and lower levels of total and defective proviral genomes, underlying the relationship between the innate immune system and the cells harbouring HIV reservoir [21]. Other studies explored the potential role of NK cells to achieve ART‐free viral remission. Two independent HIV‐1 eradication trials have shown that elevated NK load and cytotoxic responses are the major correlates of reduced levels of HIV‐1 DNA in ART patients treated with latency‐reversing agents [22, 23]. The discussed studies explored the function of NK cells in adults. The few studies that previously investigated NK cells in PHIV under ART produced discordant results, likely due to differences in clinical and viral histories.

Nonetheless, according to current knowledge, ART does not fully reconstitute NK phenotype and function and exact characteristics of residual NK subset abnormalities in treated PHIV require further investigation. In this study, we investigated the NK compartment in a homogenous group of PHIV who initiated ART within the first 24 months of life and maintained continuous viral suppression since the first viral control for at least five years [24].

## 2 Methods

### 2.1 Ethics statement

This study was approved by the local institutional ethics committees of Bambino Gesù Children's Hospital (Rome, Italy), University of Padua (Padova, Italy), University Hospital 12 de Octubre and Hospital Gregorio Marañón (Madrid, Spain), St Mary's University (Twickenham, UK), Great Ormond Street Hospital (London, UK), Brighton and Sussex University Hospitals (Brighton, UK). Study participants or their legal guardians gave written informed consent in accordance with the Declaration of Helsinki.

### 2.2 Study participants

Forty PHIV, who started ART within the first two years of life, were recruited cross ‐sectionally in CARMA (Child and Adolescent Reservoir Measurements on early suppressive ART). This protocol is part of the existing EPIICAL consortium (Early treated Perinatally HIV‐Infected individuals: Improving Children's Actual Life) [25]. Inclusion criteria were as follows: (1) ≥5 years of age, (2) viral suppression (<400 copies/mL) achieved in the first year of ART and maintained for at least five years, and (3) plasma viral load of <50 HIV‐1 RNA copies/mL at enrolment. Characteristics of participants are described in Table 1. For NK experiments, two out of 40 enrolled patients were excluded for unavailability of the sample.

**Table 1:** Characteristics of the study population

	LT (n = 9)	ET (n = 29)	*p* values^a^
Male/female (N)	3/6	9/20	1.0000
At ART start			
Age (days)	571 (254.5 to 630.5)	110 (6.5 to 140)	**<0.0001**
%CD4^+^ T cells	24 (17 to 35)	31 (20 to 43)	0.4028
Plasma HIV RNA (copies/µL)	181.1 (55.6 to 620)	110 (10.2 to 500)	0.7295
Time to suppression (months)	1.6 (0.4 to 2.8)	2.5 (0.3 to 5.2)	0.3119
At analysis			
Age (years)	9 (7.5 to 17.5)	13 (9 to 16.5)	0.4706
% CD4^+^ T cells	42 (34 to 46)	39 (31.5 to 47)	0.7872
Plasma HIV RNA (copies/mL)	<40	<40	
HIV DNA (copies/10^6^ PBMCs)	112.4 (48.5 to 260.6)	33.1 (0 to 74.7)	**0.0057**
Time on ART (years)	8 (6 to 16)	13 (9 to 16)	0.2643
CMV serology (N positive/negative)	8/1	20/9	0.3958

Values are expressed as medians (interquartlile range, IQR) except number of individuals (N). CMV, cytomegalovirus. Clinical and demographic characteristics of the CARMA cohort divided into Early Treated patients (ET) and Late Treated patients (LT). *p* values in bold reached statistical significance (<0.05).

^a^ Calculated by Mann–Whitney U‐test; for categorical variables, Fisher's exact test was used.

### 2.3 Sample collection and NK FACS analyses

**Figure 1:**
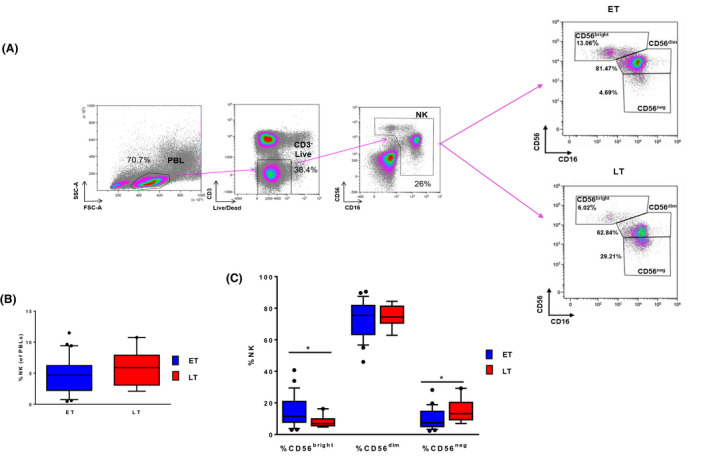
**(A)** The gating strategy to identify natural killer (NK) cells and their CD56^bright^, CD56^dim^ and CD56^neg^ subsets is shown in ET versus LT. **(B)** Frequency of total NK in ET versus LT. **(C)** Frequency CD56^bright^, CD56^dim^ and CD56^neg^ comparing ET and LT. In blue ET, in red LT.

**Figure 2:**
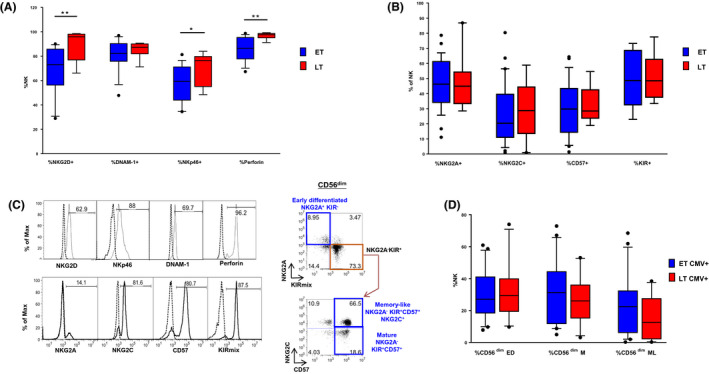
**(A)** Frequency of NK expressing NKG2D, DNAM‐1, NKp46 and perforin. **(B)** Frequency of NK expressing NKG2A, NKG2C, CD57 and KIR in CMV+ patients. **(C)** Histograms showing the expression of NKG2D, NKp46, DNAM‐1, perforin, NKG2A, NKG2C, CD57 and KIRmix on NK (open histograms) as well as control IgG signal (dashed lines) are shown in the left panel. The gating strategy used to identify different maturation subsets (Early differentiated (ED), Mature (M) and Memory‐like (ML)) based on NKG2A, NKG2C, CD57 and KIR expression among gated CD56^dim^ cells is shown on the right panel. **(D)** Distribution of CD56^dim^ ED, CD56^dim^ M and CD56^dim^ ML in CMV+ patients. In blue ET, in red LT.

Peripheral blood mononuclear cells (PBMCs) were isolated from EDTA‐anticoagulated blood using Ficoll and centralized at the University of Rome “Tor Vergata.” To assess viability, PBMCs were stained with the LIVE/DEAD fixable NEAR‐IR dead cell stain kit (Life Technologies, Carlsbad, CA, USA). Further, they were surface stained before undergoing permeabilization for intracellular staining using FOXP3 Fix/Perm Buffer Set (Biolegend); all antibodies are listed in Table S1. Conjugated mouse IgG was used for isotype control (BD Pharmingen, San Diego, CA, USA). After staining, cells were acquired on Cytoflex (Beckman Coulter, Brea, CA, USA) or, for cytotoxicity assay on FACSCanto II (BD Biosciences, Franklin Lake, NJ, USA). Gating strategies are provided in Figures 1A and 2D. Data analyses were performed using FlowJo (TreeStar, Ashland, OR, USA) or Kaluza software (Beckman Coulter).

### 2.4 NK cytotoxicity assay

A flow cytometry‐based degranulation assay was performed to measure the level of anti‐HIV‐1 cytotoxicity. We used patients' PBMCs as effectors (E) and K562 cells as target (T) cells at an E:T ratio of 10:1 and measured the frequency of CD107a^+^ NK degranulating as previously described [26].

### 2.5 IFN‐γ production by NK

IFN‐γ accumulation in NK was analysed by flow cytometry after 20 hours of PBMC stimulation with or without cytokines (5 ng/mL IL‐12, 100 ng/mL IL‐15, 50 ng/mL IL‐18; Peprotech, London, UK), as described previously. [26], where PBMCs were cultivated for 20 hours.

### 2.6 Quantitative total HIV‐1 DNA assay

Total HIV‐1 DNA was isolated from PBMCs and quantified by real‐time quantitative reverse transcription PCR (qRT‐PCR) as previously described [27]. All measurements were done in triplicates. The results are reported as HIV‐1 copies/10^6^ PBMCs.

### 2.7 RNA‐seq

Total RNA was extracted (QIAGEN RNeasy Plus Mini Kit) from cryopreserved PBMC. All samples generated high quality RNA (RIN 7.9 – 9.8, mean 9.3). Total stranded RNA libraries were prepared using Roche Kapa RNA Hyper with HMR ribodepletion according to manufacturer's standard protocol. 20 ng RNA was used as input with 14 cycles of library amplification. Libraries were balanced and sequencing on a NovaSeq 6000 S1 200 cycle V1 flow cell to generate 50M PE100 reads per sample. The raw demultiplexed FASTQ paired‐end read files were trimmed of adapters and filtered using the program skewer to remove any with an average Phred quality score of <30 or a length of <36 bp. Aligned reads were counted and assigned to gene meta‐features using the program featureCounts as part of the Subread package. Count files were assessed for quality control, normalized and analysed using an in‐house pipeline using the LIMMA‐trend method for differential gene expression testing and the gene set variation analysis (GSVA) library for GSVA as previously described [28, 29] (Chen EY, BMC Bioinformatics, 2013). All raw RNA‐Seq data are available on the Gene Expression Omnibus: NCBI gene expression and hybridization array data repository (GEO) GSE168658.

### 2.8 Statistical analyses

Intergroup comparisons were performed using Mann–Whitney U test for non‐normal variables or unpaired t‐test for normal variables. Normality was assessed using Shapiro–Wilk test. Paired comparisons were made using nonparametric Wilcoxon's test or parametric t*‐*test. Fisher's exact test was used for categorical variables. To describe the association between each NK subset and HIV‐1 DNA, a multivariable Poisson regression model was performed. The viral load and %CD4^+^ T‐cells at the time of ART start and age at ART start (ET vs. LT) as interaction term were selected as confounders by stepwise AIC variable selection. Statistical analyses were performed using GraphPad Prism 6.0 (San Diego, CA) and R Bioconductor. To visualize interactions, sjPlot R package was used. The threshold for statistical significance was set to *p* < 0.05. The R package “enrichR” v2.1 was used to perform gene ontology and pathway enrichment analysis in Reactome 2016, WikiPathways 2019 (Human), BioCarta2016 and KEGG2019 (Human) databases [30, 31]. NK‐cell‐mediated cytotoxicity pathway was created using the KEGG online tool KEGG Mapper – Search&Color Pathway.

## 3 Results

### 3.1 Study cohort

Age at ART initiation was used to stratify the cohort. According to previous data [6, 7, 8, 9] the six months cut‐off was chosen, resulting in patients treated ≤6 months (Early Treated patients, ET; n = 29) and >6 months (Late Treated patients, LT; n = 9) (Table 1). ET and LT presented similar CD4^+^ T‐cells and plasma HIV‐1 RNA load at ART initiation, time to viral suppression and male/female ratio. At the time of NK analyses, ET and LT did not differ significantly for median age, ART duration, %CD4^+^ T cells, HIV‐1 RNA, CMV^+^ serology (see Table 1) and race. Conversely, HIV‐1 DNA burden was significantly lower in ET compared with LT (median 33.1 vs. 112.4 copies/10^6^ PBMCs, *p* = 0.0057).

### 3.2 LT present a perturbation in NK subset distribution compared with ET despite long‐term viral control

The two groups showed similar frequency of total NK (Figure 1B), yet the analysis of their distribution according to CD56 and CD16 expression showed a significantly higher proportion of CD56^neg^ (15 ± 7% vs. 10 ± 6%, *p* = 0.045) associated with a lower proportion of CD56^bright^ cells (8 ± 4% vs. 15 ± 10%, *p* = 0.031) in LT compared with ET (Figure 1C). We next evaluated whether ET and LT differed for expression of the main NK markers including activating (i.e. NKG2D, DNAM‐1, NKp46, NKG2C) and inhibitory (i.e. NKG2A, KIRs) receptors, perforin and the CD57 terminal differentiation marker (Figure 2A,B). Furthermore, we evaluated the expression of NKG2D, NKp46, DNAM‐1, perforin, NKG2A, NKG2C, CD57 and KIRmix (KIR2DL1/S1/S3/S5 and KIR2DL2/L3/S2) on NK (Figure 2C, left panel). When compared with ET, LT displayed a significantly higher frequency of NKG2D^+^ NK (90 ± 13% vs. 68 ± 21%, *p* = 0.008) and an increase in the proportion of NKp46^+^ NK (71 ± 13% vs. 57 ± 15%, *p* = 0.05) and %perforin^+^ (97 ± 3% vs. 86 ± 10%, *p* = 0.010) (Figure 2A). No differences were found in the frequency of NKG2A, NKG2C, CD57 and KIR on NK between LT and ET (Figure 2B). Moreover, we analysed the distribution of CD56^dim^ cells in maturation subsets, which progress from early‐differentiated (ED; NKG2A^+^KIR^−^CD57^−^) to fully mature (M; NKG2A^−^KIR^+^CD57^+^) and, eventually, to memory‐like (ML; NKG2A^−^NKG2C^+^KIR^+^CD57^+^) (Figure 2C, right panel). Of note, ML is a highly cytotoxic NK subset with immune adaptive properties that is expanded in CMV‐seropositive individuals and, to a higher extent, in HIV/CMV co‐infected patients [32, 33]. This analysis did not reveal significant differences between ET and LT when excluding CMV‐seronegative patients (Figure 2D) versus not (data not shown), although there is a trend in ET CMV+ to express higher %ML compared with LT.

### 3.3 Evaluation of NK functionality in ET and LT and association with HIV‐1 DNA

**Figure 3:**
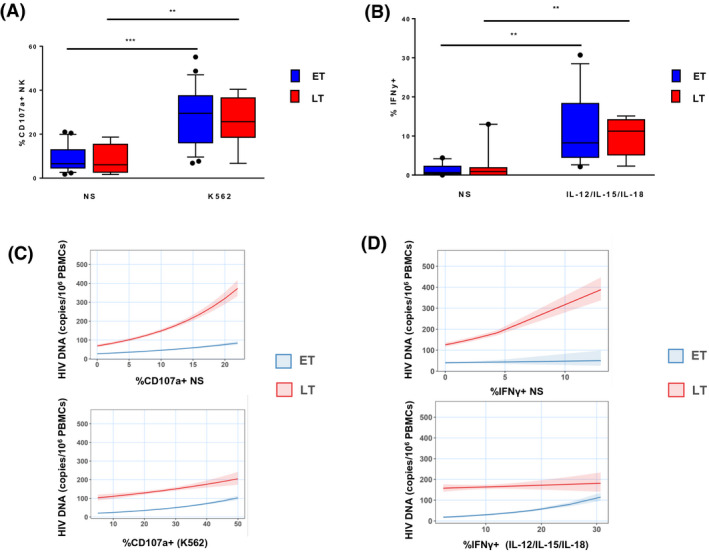
**(A)** Frequency of NK expressing CD107a in cells not‐stimulated (ns) or stimulated with K562 target in ET versus LT. **(B)** Frequency of NK expressing IFN‐γ in cells not‐stimulated or stimulated with a cytokine cocktail (IL‐12, IL‐15, IL‐18) in ET versus LT. **(C)** Multivariable Poisson regression model showing the association between CD107a+ NK ns or stimulated with K562 target with HIV reservoir in ET versus LT. **(D)** Multivariable Poisson regression model showing the association between IFN‐γ+ NK ns or stimulated with cytokines with HIV reservoir in ET versus LT.

Next, we performed a comparative evaluation of the functional activity of NK in ET and LT. Both groups responded significantly to stimulation with K562 target cells (*p* = 0.001 and *p* = 0.0074 respectively) (Figure 3A). No differences in terms of NK cytotoxicity were found among the groups. We also found a positive correlation between spontaneous degranulation and %CD56^neg^ (data not shown) in LT. The analysis of NK IFN‐γ response shows that both groups significantly responded to cytokine stimulation (*p* = 0.0015 and *p* = 0.0079 respectively), but no differences were found between the groups (Figure 3B). Conversely, NK functionality and the size of the viral reservoir showed different levels of association in ET and LT. NK that spontaneously expressed CD107a were positively associated with the size of HIV‐1 DNA reservoir in both LT and ET: for each 1% increase CD107a^+^ ns cells, the HIV‐1 DNA was increased by 8% in LT (Figure 3C). This association was decreased by 3% in ET, showing a less pronounced slope than LT. Furthermore, a difference between ET and LT was also found between IFN‐γ and the size of HIV‐1 DNA. In LT we found that, for each 1% increase IFN‐γ^+^ ns cells, the HIV‐1 DNA was increased by 9%. However, this association was weaker in ET compared to LT and decreased by 7% (Figure 3D).

### 3.4 NK features differently associate with the HIV‐1 DNA load in LT compared with ET

**Figure 4:**
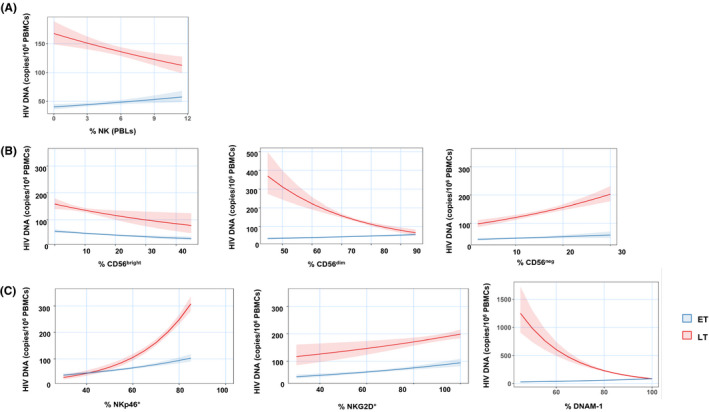
Multivariable Poisson regression model showing the association between the HIV reservoir and **(A)** % NK (of PBLs); **(B)** CD56^bright^; CD56^dim^; CD56^neg^; **(C)** %NKp46; %NKG2D; %DNAM‐1 in ET versus LT.

We further investigated the association of multiple NK subsets with HIV‐1 DNA (Table 2). According to the multivariable regression model, the frequency of total NK (Figure 4A), %CD56^dim^ (Figure 4B, middle panel) and %DNAM‐1^+^ NK (Figure 4C, right panel) was inversely associated with HIV‐1 DNA size in LT, but positively associated in ET. A distinct association in the two groups was also found between the frequency of CD56^neg^ (Figure 4B, right panel) and NKp46^+^ NK (Figure 4C left panel) with HIV‐1 DNA. Indeed, the association between these subsets and HIV‐1 DNA in ET was less pronounced (Table 2) than in LT. No significant differences were found between ET and LT in the association of HIV‐1 DNA with %CD56^bright^ (Figure 4B left panel) or %NKG2D (Figure 4C middle panel) or %CD56^dim^ maturation subsets and HIV‐1 DNA burden (Table 2).

**Table 2:** A unit change in the NK parameter is associated with the indicated % change in HIV‐1 DNA (e.g. 1‐unit increase in NK cell % of peripheral blood lymphocytes (PBLs) is associated with 4% decrease in HIV DNA considering all patients and this effect is 6% higher in ET)

NK parameters	LT					ET			
	N	N	% Diff	β (95% CI)	*p* *	N	% Diff	β (95% CI)	*p* *
% of PBLs	38	9	−4	0.96 (0.94 to 0.98)	**0.0004**	29	6	1.06 (1.04 to 1.10)	**3.78 × 0^−06^**
CD56^bright^ %	38	9	−2	0.98 (0.97 to 0.99)	**0.001**	29	0.06	1.0006 (0.99 to 1.01)	0.930
CD56^dim^ %	38	9	−4	0.96 (0.96 to 0.98)	**8.25 × 10^−11^**	29	4	1.04 (1.03 to 1.06)	**3.86 × 10^−12^**
CD56^neg^ %	38	9	3	1.03 (1.02 to 1.04)	**1.67 × 10^−42^**	29	−1	0.99 (0.97 to 0.99)	**0.002**
NKG2D^+^ %	23	7	1	1.01 (1.00 to 1.01)	**0.004**	16	0.04	1.0004 (0.99 to 1.01)	0.276
NKp46^+^ %	23	7	4	1.04 (1.04 to 1.05)	**1.75 × 10^−42^**	16	−2	0.98 (0.97 to 0.98)	**2.47 × 10^−10^**
DNAM‐1^+^ %	23	7	−5	0.95 (0.95 to 0.96)	**1.77 × 10^−31^**	16	6	1.06 (1.06 to 1.08)	**6.83 × 10^−37^**
Perforin^+^ %	23	7	−11	0.89 (0.86 to 0.90)	**1.72 × 10^−30^**	16	11	1.11 (1.09 to 1.14)	**1.24 × 10^−21^**
Perforin MFI × 10^−3^	23	7	0.01	1.0001 (1.0001 to 1.0001)	**7.05 × 10^−46^**	16	−1	0.99 (0.99 to 0.99)	**6.28 × 10^−63^**
% of CD56^dima^									
Early differentiated	28	8	2	1.02 (1.02 to 1.02)	**1.40 × 10^−32^**	20	0	1.00 (0.99 to 1.00)	0.662
Mature	28	8	−3	0.97 (0.97 to 0.98)	**8.54 × 10^−27^**	20	0.3	1.003 (0.996 to 1.01)	0.379
Memory‐like	28	8	0.1	1.001 (0.996 to 1.01)	0.561	20	−3	0.97 (0.96 to 0.98)	**7.80 × 10^−14^**
CD107a^+^ %									
ns	35	8	8	1.08 (1.07 to 1.09)	**4.10 × 10^−67^**	27	−3	0.97 (0.96 to 0.99)	**3.78 × 10^−05^**
+K562	35	8	2	1.02 (1.01 to 1.02)	**7.77 × 10^−06^**	27	2	1.02 (1.01 to 1.03)	**1.69 × 10^−06^**
IFNγ^+^ %									
ns	22	9	9	1.09 (1.08 to 1.10)	**4.30 × 10^−40^**	13	−7	0.93 (0.88 to 0.99)	**0.023**
+IL‐12/IL‐15/IL‐18	22	9	0.4	1.004 (0.99 to 1.02)	0.425	13	6	1.06 (1.04 to 1.08)	**3.96 × 10^−12^**

The β estimates adjusted for the age groups (ET/LT), % CD4+ T‐cells, and HIV RNA copies/µL at baseline. The % CD107a+ and IFN‐γ+ NK cells were evaluated in functional assays described in Figure 3. % Diff, difference in total HIV DNA (copies/10^6^ PBMCs). β, regression coefficient (95% CI, confidence interval).

^a^ Only CMV+ patients were considered to test association with CD56^dim^ maturation subsets. ns, non‐stimulated. N= number of patients analysed.

* *p* values in bold reached statistical significance (<0.05).

### 3.5 RNA‐seq analyses confirm that LT display a more activated profile with increased cytotoxic function compared with ET

**Figure 5:**
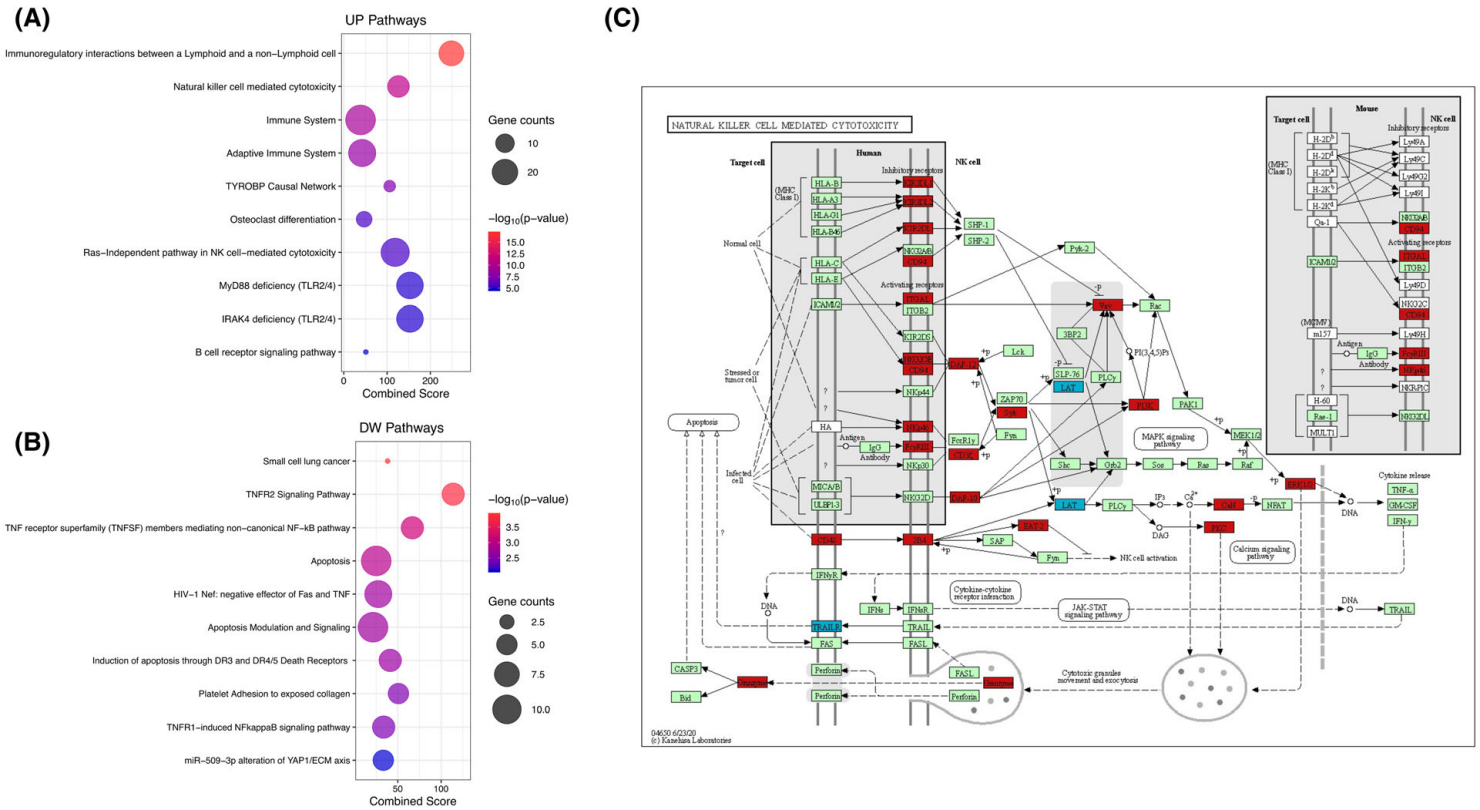
Pathway analysis applied to **(A)** differentially expressed genes (DEGs) up‐regulated (UP) and **(B)** DEGs down‐regulated (DW) in ET versus LT patients. **(C)** Natural killer cell mediated cytotoxicity pathway depicting in red up‐regulated DEGs and in blue down‐regulated DEGs with a significant *p*‐value (<0.05) in ET versus LT.

To explore the impact of timing of ART initiation on gene expression, we performed regression analysis using RNA‐Seq on PBMCs from 23 patients of the CARMA cohort (16 ET and 7 LT). Overall, we found 388 differentially expressed genes (DEGs) between LT and ET. We found 179 up‐regulated (UP) and 209 down‐regulated (DW) genes in LT vs. ET (*p* < 0.05). We also applied regression to the PBMC transcriptional dataset using the proportion of total NK in order to correlate a gene expression signature with total NK levels, identifying 1,167 DEGs between LT and ET. Among these, 660 genes were UP, whereas 507 genes were DW (*p* < 0.05). To understand the potential biological roles of these DEGs, we performed pathway enrichment analyses. The top 10 significantly enriched pathways (UP and DW) are shown in Figure 5A,B and this also included NK‐mediated cytotoxicity pathway (WikiPathways, 2019) (Figure 5C). The UP genes in LT were mainly involved in immune‐regulatory interactions (CD160, CD300LB, KLRF1, SIGLEC7, SLAMF6) and NK‐mediated cytotoxicity (CD48, CD244, GZMB, HCST, KIR3DL1, KIR3DL2, KIR2DL3, KIR2DL4, MAPK1, PIK3R1, SH2D1B, TYROBP, VAV3) (Figure 5A).

In particular, CD160, expressed mainly by the CD56^dim^ subsets, is involved in NK cytotoxic activity. Related to this function, Huth et al. described that the activation of proteins of the MAPK pathway leads to intracellular signals that cause the release of cytokines from NK cells and activate cytotoxicity [34, 35]. The up‐regulation of several genes involved in activating signals of the NK subsets (TYROBP, CD300LB, KIR3DL1, KIR3DL2, KIR3DL3, KIR3DL4 and GRZB) in LT compared with ET, confirms the increased activation of NK found in these patients (Figure 2A), leading, in agreement with our results, to a higher expression of CD56^neg^ NK (Figure 1C).

### 3.6 NK cells are more susceptible to apoptosis in LT compared with ET

Pathway analysis applied to DW genes in LT versus ET also highlighted genes related to apoptosis and TNFR signalling pathway, two pathways that displayed some significant genes in common (BIRC2, TRAF1, TRAF2). BIRC2 protein inhibits apoptosis through the binding to TRAF1 and TRAF that are TNFR‐associated factors. When it is DW, as in the case of our LT, the apoptosis is not inhibited, and cells go through programmed death.

## 4 Discussion

This study seeks to provide a description of the NK population in PHIV and it adds on the previous literature showing an impact of timing of ART initiation on NK subset distribution in PHIV despite long‐term viral control due to effective ART. We found that the distribution of NK into the major CD56^bright^, CD56^dim^ and CD56^neg^ subsets was different according to the time of ART initiation, with LT showing a higher proportion of CD56^neg^ cells and a lower proportion of CD56^bright^ cells when compared with ET. The CD56^neg^ subset typically expands in chronic HIV‐1 infection regardless of antiviral treatment and consists of a highly dysfunctional NK subset with impaired effector functions, produces a low amount of anti‐viral cytokines/chemokines and releases regulatory cytokines that suppress IFN‐γ release by CD8^+^ T‐cells, contributing to the poor immune control over HIV‐1 and to disease progression [12, 36, 37]. It is commonly believed that CD56^neg^ cell expansion results from the loss of CD56 expression by persistently stimulated NK in prolonged immune activation and inflammatory processes established in chronic HIV‐1 infection [38]. Few studies in PHIV have identified some limited NK phenotypic changes, which persist despite treatment [39, 40]. One study examined 16 PHIV during their first year of life, showing that the NK compartment was comparable to healthy controls at birth, then NK frequency progressively declined in parallel with CD56^neg^ cell expansion, but data following ART initiation were missing [41]. Here we show that only LT PHIV present a high frequency of dysfunctional CD56^neg^ NK, whereas ET display a normal NK subset distribution that is comparable to that of healthy individuals (data not shown). To the best of our knowledge, this study on PHIV is the first providing evidence that timing of ART initiation, more than a prolonged viral suppression, has an impact on the long‐term recovery of a physiological NK compartment, which is crucial for the innate immune defence against HIV‐1 as well as other pathogens or tumours [42].

Additionally, our results showed that delayed ART start is associated with significantly higher expression of NKG2D, perforin and NKp46, that may reflect an increased activation status of NK in LT compared with ET. It is well‐established that HIV‐1 infection induces chronic immune activation and systemic inflammation that may in part persist despite successful ART‐mediated viral suppression as measured by an enhancement of microbial translocation at mucosal membranes, inflammatory cytokine production and activation of immune cells, including NK [43]. Increased NK activation was also found at birth in PHIV, possibly due to *in utero* exposure to inflammatory stimuli [41]. Therefore, we suppose that delayed time of therapy initiation might have been less effective at normalizing the activation status of NK in LT. Potential mechanisms behind persistent immune activation in this group include reactivation of infections such as CMV (more than 85% of LT were CMV seropositive). Indeed, it has been previously shown that CMV intervention is able to reduce T‐cell activation in HIV‐1‐positive patients [44]. Similarly, the destruction of the mucosal membrane, which occurs during the early phase of the infection, could change gut microbiota composition and release microbial products that activate innate immunity promoting inflammation, further driving systemic activation [44, 45].

Notwithstanding the increased proportion of dysfunctional CD56^neg^ cells in LT, the NK cytotoxicity against K562 target cells was comparable in ET and LT. This apparent incongruence may be related to the fact that K562 cells are predominantly recognized and killed by NK via their NKG2D receptor, whose expression is enhanced on LT NK, possibly counterbalancing the presence of anergic CD56^neg^ cells. NK of both groups had also comparable IFN‐γ responses following cytokine stimulation, thus showing similar overall functionality, at least on the basis of generic *ex vivo* tests.

Multivariable regression analyses highlighted some major differences between ET and LT in the relationship of some NK features with the HIV‐1 reservoir. Specifically, the frequency of the dysfunctional CD56^neg^ subset, of NK expressing NKp46 and, in unstimulated conditions, CD107a and IFN‐γ, were significantly inversely associated with the HIV‐1 DNA reservoir burden in ET, whereas a direct correlation was found in LT. These NK features, except for %CD107a^+^ and %IFN‐γ^+^ non‐stimulated NK, were significantly higher in LT, who also had higher HIV‐1 DNA as compared with ET. In addition, regression analyses on RNA‐seq of NK markers revealed as LT present a higher susceptibility to apoptosis, cytotoxic function and activation compared with ET. Therefore, these results suggest that a later ART start, previously described to be related to an increased immune activation of the T‐cell compartment [46], can also drive chronic NK activation. Apparently, this condition is subverted in ET who had a smaller HIV‐1 reservoir, that decreased with an increase of anergic, NKp46^+^, perforin^high^ and basally activated NK. Also, the multivariable regression analysis showed that frequencies of total and CD56^dim^ NK were directly associated with HIV‐1 DNA in ET but inversely in LT, supporting a preservation of the functional NK compartment if ART is started early, before virus‐induced deregulation of NK has occurred [47]. Finally, we found that ET and LT also differed in the interaction between DNAM‐1^+^ NK and HIV‐1 reservoir, with a positive and inverse association in ET and LT respectively. Previous studies have shown that DNAM‐1, together with NKG2D and NKp46, mediates in vitro NK recognition and killing of HIV‐1‐infected T cells [48] and that increased frequency of DNAM‐1^+^ NK could be found in some cohorts of HIV‐1‐positive patients [39, 49]. However, as opposed to NKG2D and NKp46, DNAM‐1 is similarly expressed on NK of ET and LT, hence the significance of differential association with HIV‐1 reservoir in the two groups is at present unclear. It remains unknown whether the long‐term preservation of NK distribution and function may protect PHIV from higher viral reservoir seeding in case of viral rebound.

Our study has some limitations. First, this is a cross‐sectional analysis of reservoir, thus subjected to causality. The reduced number could represent a weakness, which likely diminishes the prediction capacity of all the models. However, this reflects the clinical reality: a small number of children started ART in infancy, achieve and consistently maintain viral control for many years. Nonetheless, this paper relies on a well‐characterized homogenous cohort of PHIV, in which we could demonstrate that starting ART within the first six months of age has long‐lasting beneficial effects on the NK compartment. This mainly consists of a reduced immune activation and a better functionality of the NK compartment.

## 5 Conclusions

Our data suggest that early ART preserves the NK compartment. Further work is needed to define the extent to which timing of ART start affects the potential capacity of NK against latent HIV‐1 in PHIV and if a booster strategy can be used to achieve a better suppression of viral reservoir in these patients through NK pathways.

## Competing interests

The authors have declared no conflict of interest.

## Authors’ contributions

P.P., N.C., M.D. and S.Z. designed the study; M.D., S.Z., K.G. and S.R. performed the experiments; M.D., S.Z., S.D.R., M.K., B.R., A.R., S.L.W. and G.R.P. analysed the data; M.D., S.Z. and P.P drafted the manuscript. A.T., P.R.C., E.N., C.F., A.D.R. and C.G. provided patients’ samples. P.P, P.R., S.P., E.N. and P.R.C. supervised the study. All the authors read and approved the manuscript.

## Funding

This study was supported by PENTA‐ID Foundation (http://penta‐id.org/), funded through an independent grant by ViiV Healthcare UK. The funders had no role in study design, data collection and analysis, decision to publish, or preparation of the manuscript. This work was also supported by grants of the Italian Ministry of Health to M.D. (Ricerca Finalizzata RF‐2016‐02363642) and the National Institute of Health (NIH) to P.P. (5U01AI135941‐04).

## EPIICAL Consortium

Mark Cotton, Shaun Barnabas, Nigel Klein, Thanyawee Puthanakit, Louise Kuhn, Andrew Yates, Avy Violari, Kennedy Otwombe, Paula Vaz, Maria Grazia Lain, Tacilta Nampossa, Denise Naniche, Sheila Fernandez‐Luis, Elisa Lopez, Philip Goulder, Holly Peay, Rajendra Pahwa, Katherine Luzuriaga, Ofer Levy, Kinga Smolen, Moira Spyer, Vincent Calvez, Anne‐Geneviève Marcelin, Maria Angeles Munoz, Annalisa Dalzini, Deborah Persaud, Nicolas Chomont, Mathias Lichterfeld, Silvia Faggion, Daniel Gomez Pena, Andrea Oletto, Alessandra Nardone, Paola Zangari, Carla Paganin,William James, Inger Lindfors, Shrabon Samiur Hassan, Francesca Mazzetto, Hellen Akisinku, Musakanya Chingandu, Francesca Rocchi, Ilaria Pepponi, Rob J De Boer, Juliane Schröter, Viviana Giannuzzi, Andrew Yates, Sinead Morris.

## Supporting information


**Table S1**. List of antibodies.
