# Supplementary material for: Early ART initiation during infancy preserves natural killer cells in young European adolescents living with HIV (CARMA cohort)
Source: J Int AIDS Soc. 2021 Jul 8;24(7):e25717. doi: 10.1002/jia2.25717 (PMC8264399; doi:10.1002/jia2.25717)
Supplement: Supplementary file 1 — Table S1. List of antibodies. [file JIA2-24-e25717-s001.docx]

**Supplementary Table 1. List of Antibodies.**

| **Antibody** | **Fluorophore** | **Clone** | **Manufacturer** |
| --- | --- | --- | --- |
| **Surface staining** | | | |
| CD3 | AF700 | UCHT1 | BD |
| CD3 | APC | UCHT1 | Biolegend |
| DNAM-1 (CD266) | FITC | 11A8 | Biolegend |
| NKp46 | PE-Cy7 | 29A1.4 | Biolegend |
| KIR2DL1/S1/S3/S5 | APC | HP-MA4 | Biolegend |
| KIR2DL2/L31/S2 | APC | 16A11 | Biolegend |
| NKG2D (CD134) | PE | 1D11 | Ebioscience |
| NKg2A (CD159a) | FITC | S19004C | Miltenyi |
| NKg2C (CD159c) | PE | S19005E | Miltenyi |
| CD16 | BV510 | 3G8 | BD |
| CD16 | APC-e Fluor780 | 3G8 | Ebioscience |
| CD56 | PerCP | MEM-188 | Thermo Scientific |
| CD56 | PerCP-Cy5.5 | B159 | BD |
| CD57 | PE-Cy7 | TBO1 | eBioscience |
| CD107a (LAMP-1) | FITC | eBioH4A3 | eBioscience |
| **Intracellular staining** | | | |
| IFNy | FITC | 552887 | BD |
| Perforin | BV421 | δG9 | BD |
